# Editorial: Multimodality imaging in the evaluation of valvular heart disease

**DOI:** 10.3389/fcvm.2022.1041129

**Published:** 2022-10-21

**Authors:** Daniel A. Morris, Sebastian Kelle

**Affiliations:** ^1^Department of Internal Medicine and Cardiology, Charité-Universitätsmedizin Berlin, Berlin, Germany; ^2^Deutsches Herzzentrum Berlin, Berlin, Germany

**Keywords:** valvular heart disease, multimodality imaging, cardiovascular magnetic resonance (CMR), echocardiography, cardiac CT

The evaluation of valvular heart disease using multimodality imaging has significantly improved in the last years and, thus, recent expert consensuses and societies guidelines have recommended the use of multimodality imaging in the evaluation of valvular heart disease (1–4). In effect, the use of cardiovascular magnetic resonance (CMR) has emerged as an accurate method to evaluate the severity of aortic, mitral, and pulmonary valve regurgitation in the setting of indeterminate findings by echocardiography (1–4). Furthermore, cardiac computed tomography (CCT) has been well-validated and recommended as a comprehensive method in the pre-evaluation of patients with indication for transcatheter aortic valve implantation (TAVI) (1, 2). Notwithstanding this, it is important to highlight that transthoracic echocardiography (TTE) remains the first and standard method in the evaluation of the severity of valvular heart disease (1–4). Likewise, transesophageal echocardiography (TEE) remains a valuable method to determine the severity of valvular heart disease in cases of suboptimal or unequivocal findings from TTE (i.e., mainly in patients with potentially severe mitral valve regurgitation) (1–4). Hence, combining these modalities (TTE, TEE, CMR, and CCT, as appropriate) allows an accurate and comprehensive evaluation of patients with valvular heart disease (Figure 1). This Research Topic on “*Non-invasive and invasive cardiovascular imaging in valvular heart disease*” provides further and potential utilities of multimodality imaging in the evaluation of valvular heart disease.

**Figure 1:**
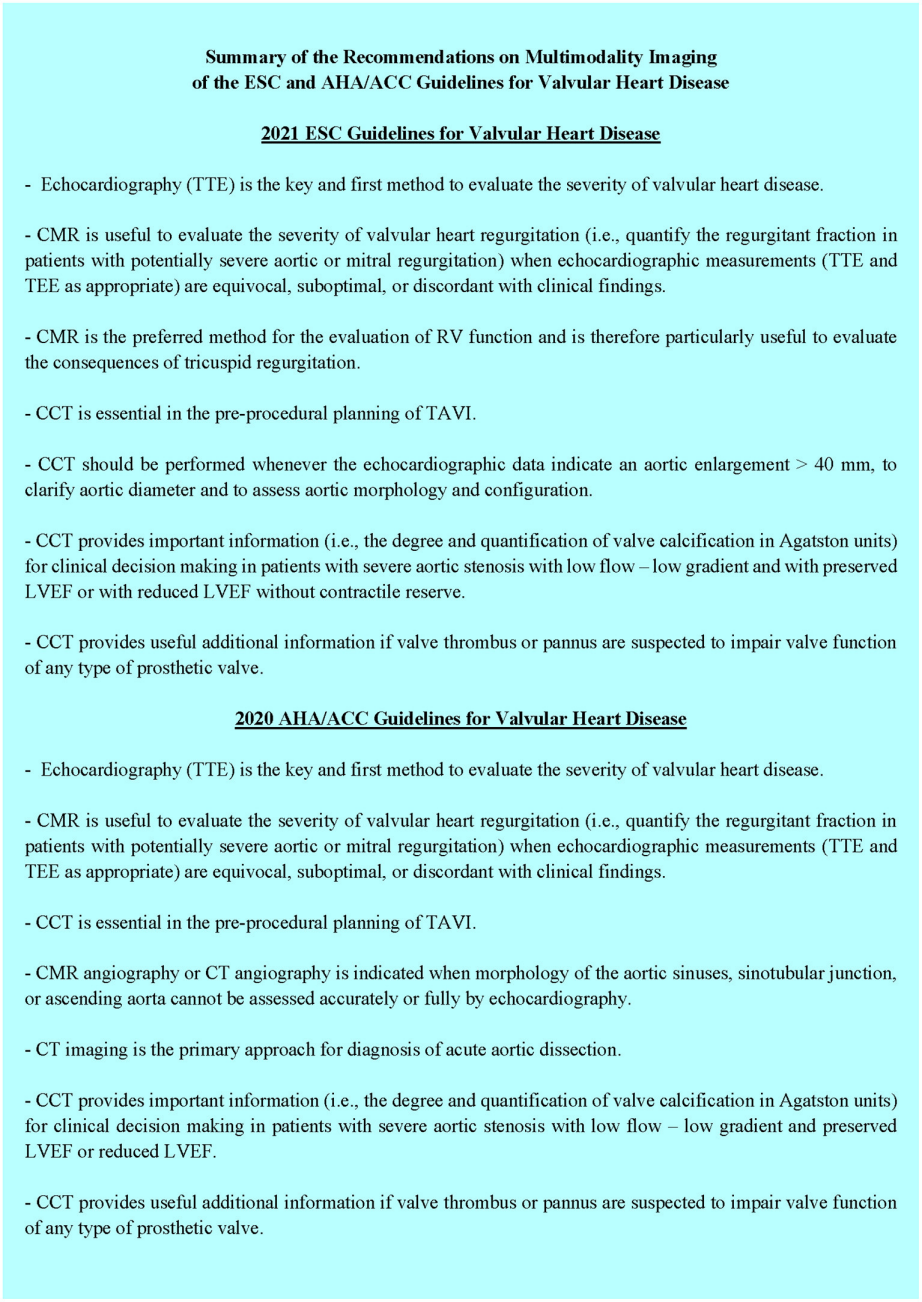
Summarized recommendations of current societies guidelines on the use of multimodality imaging in valvular heart disease.

Regarding the potential further use of CCT in patients with severe valvular heart, in this Research Topic, a research group of the Shanghai Jiao Tong University has provided new insights regarding the potential utility of CCT for the indication of an aortic valve repair in patients with severe aortic regurgitation (AR; Yang et al.). In effect, Yang et al. have shown by means of comprehensive CCT analyses and cases-videos as analyses and measurements of the aortic annulus, leaflets, and aortic root could assist in the indication and post-operative evaluation of aortic valve repair in patients with severe aortic regurgitation. In line with these findings, Hegeman et al. present in this Research Topic a comprehensive review on the use of multimodality imaging and the current surgical techniques in patients undergoing mini-invasive (transapical and port-access) mitral valve repair of severe degenerative mitral valve regurgitation (MR).

Other clinically interesting points to highlight in this Research Topic are the findings of Schneider's research group regarding the potential use of subcostal view by TTE in the evaluation of the mechanism of tricuspid valve regurgitation (TR) in patients with a ventricular pacemaker or device (i.e., CRT or ICD; Deichl et al.). In this respect, this research group of the Charité University Hospital presents an excellent review with three clear cases-examples of TR caused by cardiac implantable electronic devices. In line with these findings, Hagendorff and Stöbe present a critical and comprehensive review regarding how to accurately determine the severity of MR using TTE and TEE.

Furthermore, in this Research Topic, a group of experts have discussed, revised, and provided findings regarding the clinical relevance of arrhythmias, comorbidities, and the use of CMR in patients with aortic valve stenosis (AS; Kubala et al.; Myasoedova et al.; Mantini et al.). In this respect, a group of experts headed by Doctors Tribouilloy, Enriquez-Sarano, and Lancellotti have specially revised and discussed the difficulties in the management of severe AS and other valvular heart disease in patients with atrial fibrillation. Moreover, in this Research Topic, a research group of the Centro Cardiologico Monzino (IRCCS) has demonstrated the significant association of a sclerosed (or calcified) aortic valve (i.e., aortic valve sclerosis) with coronary artery disease (CAD; i.e., history of myocardial infarction) and worse prognosis, highlighting and suggesting that an aortic valve sclerosis could be used as a risk marker of potentially severe CAD. Furthermore, in this Research Topic, it is important to highlight the interesting findings of Mantini et al. using CMR in patients with severe AS. In this respect, this research group has proposed an interesting and practical new method using CMR (i.e., aliased orifice area planimetry by 2D phase contrast imaging) to estimate the aortic valve area in patients with AS. Likewise, in this Research Topic, another research group has highlighted the potential usefulness of CMR (using 4D phase contrast flow imaging) in patients with AR (Cesarovic et al.).

Finally, it is important to highlight in this Research Topic, the comprehensive review of Vermes et al. regarding the practical uses of CMR to evaluate the severity of native valvular regurgitation. In this respect, this group of authors comprehensively describe, analyze and discuss the medical evidence, expert recommendations, and practical considerations of CMR to determine the severity of native valvular regurgitation.

## Author contributions

All authors listed have made a substantial, direct, and intellectual contribution to the work and approved it for publication.

## Conflict of interest

The authors declare that the research was conducted in the absence of any commercial or financial relationships that could be construed as a potential conflict of interest.

## Publisher's note

All claims expressed in this article are solely those of the authors and do not necessarily represent those of their affiliated organizations, or those of the publisher, the editors and the reviewers. Any product that may be evaluated in this article, or claim that may be made by its manufacturer, is not guaranteed or endorsed by the publisher.
